# A Study on the Effects of Muscarinic and Serotonergic Regulation by *Bojanggunbi-tang* on the Pacemaker Potential of the Interstitial Cells of Cajal in the Murine Small Intestine

**DOI:** 10.7150/ijms.83986

**Published:** 2023-05-21

**Authors:** Na Ri Choi, Haejeong Jeong, Woo-Gyun Choi, Jae-Woo Park, Seok-Jae Ko, Byung Joo Kim

**Affiliations:** 1 Division of Longevity and Biofunctional Medicine, Pusan National University School of Korean Medicine, Yangsan 50612, Republic of Korea.; 2 Department of Clinical Korean Medicine, Graduate School of Kyung Hee University, Seoul 02447, Republic of Korea.; 3 Department of Gastroenterology, College of Korean Medicine, Kyung Hee University, Seoul 02447, Republic of Korea.

**Keywords:** Traditional medicine, *Bojanggunbi-tang*, gastrointestinal disease, interstitial cells of Cajal, pacemaker potential

## Abstract

In traditional Korean medicine, the 16-herb concoction *Bojanggunbi-tang* (BGT) is used to treat various gastrointestinal (GI) diseases. In this study, we investigated the regulatory mechanism underlying the influence of BGT on the interstitial cells of Cajal (ICCs), pacemaker cells in the GI tract. Within 12 h of culturing ICCs in the small intestines of mice, the pacemaker potential of ICCs was recorded through an electrophysiological method. An increase in the BGT concentration induced depolarization and decreased firing frequency. This reaction was suppressed by cholinergic receptor muscarinic 3 (CHRM3) antagonists, as well as 5-hydroxytryptamine receptor (5HTR) 3 and 4 antagonists. Nonselective cation channel inhibitors, such as thapsigargin and flufenamic acid, along with protein kinase C (PKC) and mitogen-activated protein kinase (MAPK) inhibitors, also suppressed the BGT reaction. Guanylate cyclase and protein kinase G (PKG) antagonists inhibited BGT, but adenylate cyclase and protein kinase A antagonists had no effect. In conclusion, we demonstrated that BGT acts through CHRM3, 5HTR3, and 5HTR4 to regulate intracellular Ca^2+^ concentrations and the PKC, MAPK, guanylate cycle, and PKG signaling pathways.

## Introduction

Gastrointestinal (GI) motility is controlled by the extrinsic nervous system, the intrinsic nervous system of the GI tract, and the endocrine and paracrine systems 1. Regular contractions called “slow waves” occur autonomously in GI smooth muscles and form the basis of GI movement 2. Slow-wave potentials are changes in the resting membrane potential of the GI smooth muscle that trigger the action potential; slow-wave potentials can only cause smooth muscle contraction when they reach the threshold potential that triggers the action potential 3. These electrophysiological rhythmic changes are an important key to regulating GI motility 3. However, slow waves cannot be explained by the relationship between GI smooth muscles and motor nerves alone 2. Instead, interstitial cells of Cajal (ICCs) appear to be the primary cause of slow waves 4-6. In recent decades, intensive research in animal models (e.g., mice, guinea pigs, and dogs) has revealed that ICCs act as pacemakers in the stomach, small intestine, and large intestine 7,8. Therefore, understanding ICCs has become essential to elucidate the physiology of GI motility.

Traditional medicines are imperative in treating various diseases; thus, many studies are being conducted for clinical application with traditional medicines 9. In particular, traditional medicines are receiving special attention for their good therapeutic effects in treating digestive diseases such as nausea, vomiting, diarrhea, and irritable bowel disease 10. *Bojunggunbi-tang* (BGT) is a prescription comprising 16 herbs: *Lonicera japonica, Atractylodes macrocephala, Paeonia lactiflora, Dugesia lablab, Dioscorea japonica, Crataegus pinnatifida, Poria cocos, Magnolia officinalis, Citrus unshiu, Alisma orientalis, Massa medicata, Hordeum vulgare, Zingiber officinale, Aucklandia lappa, Amomum villosum*, and *Glycyrrhiza uralensis* 11-13. Although BGT is mainly used to treat diarrhea, inflammatory bowel disease, colitis, and other GI diseases 11,12, limited scientific evidence on the effects of these herbs on GI motility exists. Therefore, we used mice as model animals to investigate the effects of BGT on the pacemaker potential of the small intestinal ICCs and the associated mechanisms.

## Materials and Methods

### Preparation of BGT

Crude BGT herbs were purchased from Kyung Hee Hanyak Co. (Seoul, Republic of Korea), and the BGT extract was prepared as described in a previous study 11. The BGT extract was obtained by boiling in distilled water at 100°C for 2 h; subsequently, it was filtered and freeze‑dried to a powdered form. Herbs and BGT mixtures were stored at the Kyung Hee University College of Oriental Medicine.

### Preparation of ICC cultures

Small intestines were excised from ICR mice (3-6 days old, both sexes). The luminal contents were removed using Krebs Ringer bicarbonate solution, tissues were pinned to the bases of Sylgard dishes, and the mucosae were removed through sharp dissection. Intestinal muscles were separated using an enzyme solution (collagenase 1.3 mg/ml [Worthington Biochemical Corporation, Lakewood, NJ, USA], bovine serum albumin 2 mg/ml [Sigma-Aldrich; St. Louis, MO, USA], and trypsin inhibitor 2 mg/ml [Sigma-Aldrich; Merck Millipore St. Louis, MO, USA]). Separated cells were cultured in a smooth muscle growth medium (Clonetics Corp., San Diego, CA, USA) at 37°C on coverslips coated with murine collagen (Falcon/BD, Swedesboro, NJ, USA). All experiments on ICCs were performed following 12 h of culture. Since ICCs have a distinct shape from other cells, they could be identified under a phase-contrast microscope. Experimental mice were ethically handled according to the Guidelines for the Care and Use of Animals approved by Pusan National University (PNU‑2022‑0120).

### Electrophysiological experiments

The cell solution comprised 5 mM KCl, 135 mM NaCl, 2 mM CaCl_2_, 10 mM glucose, 1.2 mM MgCl_2_, and 10 mM HEPES; pH was adjusted to 7.4 using NaOH. Intracellular solutions were KCl (140 mM), MgCl_2_ (5 mM), K_2_ATP (2.7 mM), NaGTP (0.1 mM), creatine phosphate disodium (2.5 mM), HEPES (5 mM), and EGTA (0.1 mM); pH was adjusted to 7.2 using KOH. The whole-cell patch-clamp method was employed to record potentials in the current clamp mode using Axopatch I-D and Axopatch 200B amplifiers (Axon Instruments, Inc., Foster, CA, USA) for electrophysiological experiments. Results were analyzed using the Axopatch ID (Axon Instruments, CA, USA) and pClamp (version 10.0). All experiments were conducted at 30°C.

### Reverse Transcription-Polymerase Chain Reaction (RT-PCR)

Total RNA was extracted using TRIzol^TM^ Reagent (Invitrogen, Waltham, MA, USA), and reverse transcription of total RNA was performed using an M-MLV cDNA Synthesis Kit (Enzynomics, Daejeon, Republic of Korea). Polymerase chain reaction (PCR) primers were as follows: the first PCR amplification with upstream primers (*Ano1*-OF, 5=-AGGCCAAGTACAGCATGGGTATCA-3= for *Ano1*; *Chrm3*-OF, 5=- AAGGCACCAAACGCTCATCT-3= for *Chrm3*; *5Htr3*-OF, 5=-ACACCATCCAGGACATCAAC-3= for *5Htr3*; *5Htr4*-OF, 5=-GATGCTAATGTGAGTTCCAACGA-3= for *5Htr4*) and downstream primers (*Ano1*-OR, 5=-AGTACAGGCCAACCTTCTCACCAA-3=for *Ano1*; *Chrm3*-OR, 5=-GCAAACCTCTTAGCCAGCGT-3=for *Chrm3*; *5Htr3*-OR, 5=-CCATGCACACCACAAAGTAG-3= for *5Htr3*; *5Htr4*-OR, 5=-GCAGCAGATGGCGTAATACCT-3= for *5Htr4*) was performed for 38 cycles under conditions of denaturing at 95°C for 30 sec, annealing at 60°C for 30 sec, and polymerization at 72°C for 1 min. The PCR products—predicted as 213 (*Ano1*), 511 (*Chrm3*), 399 (*5Htr3*), and 359 (*5Htr4*) base pairs—were separated on 1.5% agarose gel through electrophoresis.

### Statistics

All results are presented as mean ± SEM. To determine significance, data were analyzed using one-way analysis of variance, followed by Bonferroni's post-hoc test. Significance was set at *p* < 0.05. Prism version 5.0 (GraphPad, Software Inc., La Jolla, CA, USA) and Origin version 8.0 (OriginLab Corporation, Northampton, MA, USA) were used for statistical analyses.

## Results

### Effect of BGT on ICC pacemaker potential

**Figure 1:**
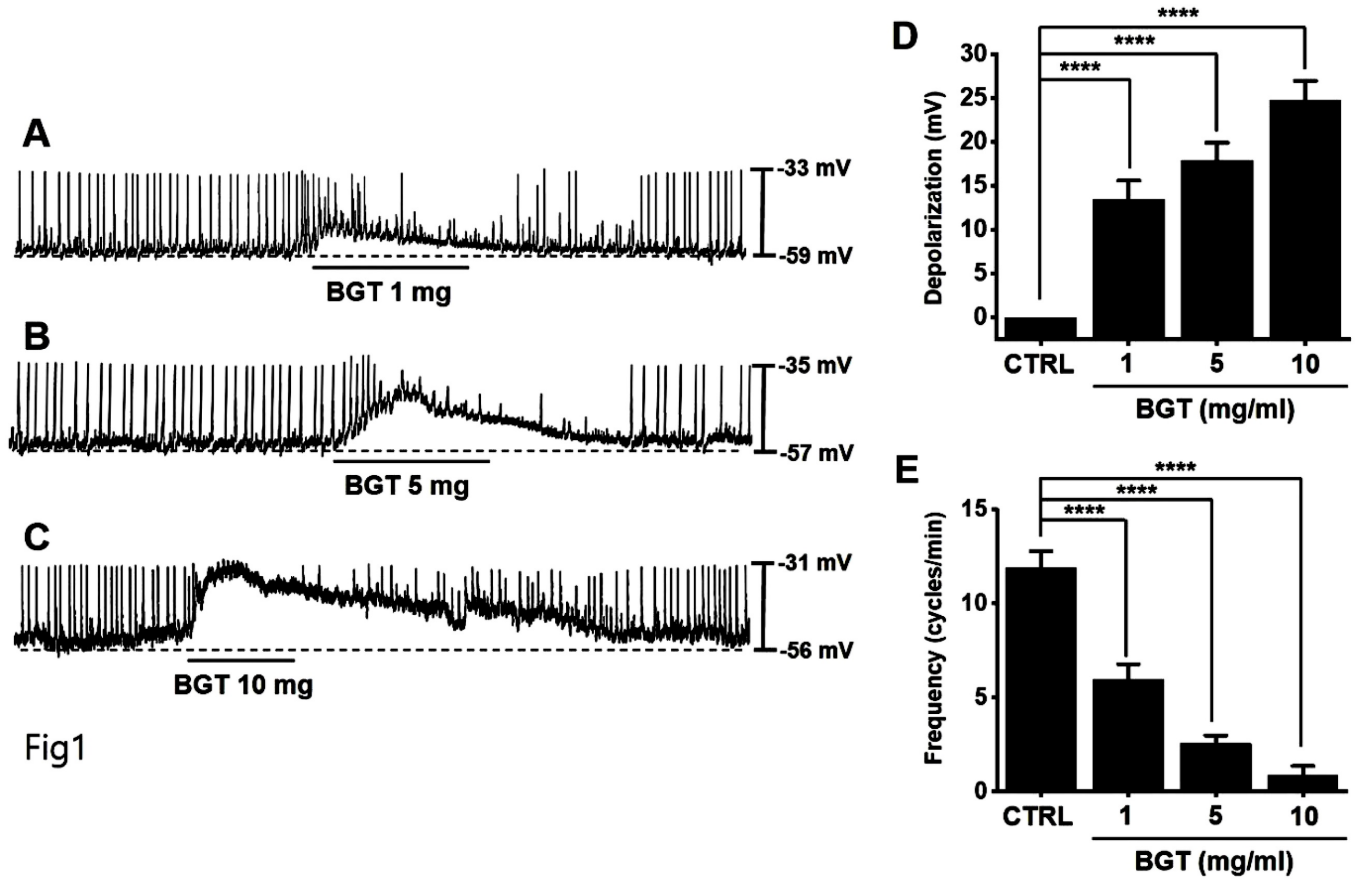
Effects of BGT on ICC pacemaker potential. (A-C) BGT depolarized pacemaker potential and reduced frequency in ICCs. (D) Bar graph for depolarization change. (E) Bar graph for frequency change. Mean ± SEM. ****p < 0.0001. CTRL, control; BGT, *Bojunggunbi-tang*.

ICCs typically showed a depolarization of 24.7 ± 2.3 mV and a frequency of 11.9 ± 0.9 cycles/min (Fig. 1). With increased BGT concentration, the pacemaker potential was depolarized, and its frequency was decreased (Figs. 1A-C). The degree of depolarization relative to 1, 5, and 10 mg/mL BGT was 13.4 ± 2.2 mV (*p* < 0.0001), 17.9 ± 2.0 mV (*p* < 0.0001), and 24.7 ± 2.3 mV (*p* < 0.0001), respectively. The frequency change across the same three BGT concentrations was 5.9 ± 0.8 cycles/min (*p* < 0.0001), 2.6 ± 0.4 cycles/min (*p* < 0.0001), and 0.9 ± 0.5 cycles/min (*p* < 0.0001), respectively. Thus, BGT depolarized the ICC pacemaker potential with increasing concentration.

### Association of muscarinic and 5HT receptors in ICC responses to BGT

**Figure 2:**
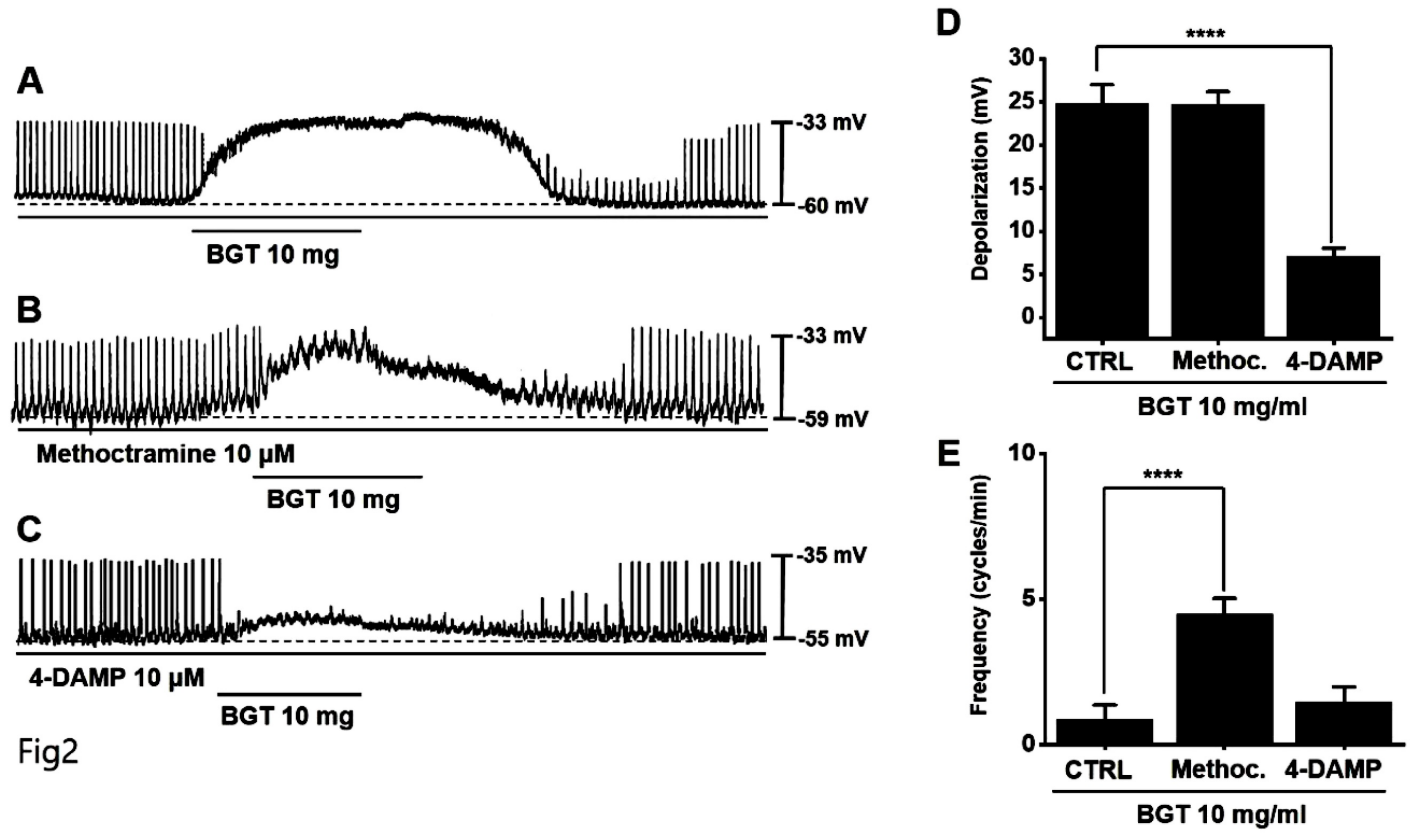
Effects of cholinergic receptor muscarinic (CHRM) antagonists on the ICC reaction to BGT. (A) 10 mg of BGT depolarized pacemaker potential and decreased frequency. (B) Methoctramine, a CHRM2 antagonist, did not affect the ICC reaction to BGT. (C) 4-DAMP, a CHRM3 antagonist, inhibited the ICC reaction to BGT. (D) Bar graph for depolarization change. (E) Bar graph for frequency change. Mean ± SEM. ****p < 0.0001. CTRL, control; Methoc., Methoctramine; BGT, *Bojunggunbi-tang*.

Muscarinic receptors are involved in regulating GI motility 14,15. As ICCs possess cholinergic receptor muscarinic (CHRM) 2 and CHRM3 16,17, we used the respective antagonists to investigate their involvement in BGT effects on ICCs. Pretreatment with the CHRM2 antagonist, methoctramine, did not alter the ICC response to BGT. Conversely, pretreatment with the CHRM3 antagonist, 4-DAMP, suppressed the response (Figs. 2A-C). Depolarization was 24.6 ± 1.5 mV under methoctramine pretreatment and 7.1 ± 1.0 mV (*p* < 0.0001) under 4-DAMP pretreatment (Fig. 2D). Furthermore, the frequency change was 4.5 ± 0.5 cycles/min (*p* < 0.0001) under methoctramine and 1.5 ± 0.5 cycles/min under 4-DAMP (Fig. 2E).

**Figure 3:**
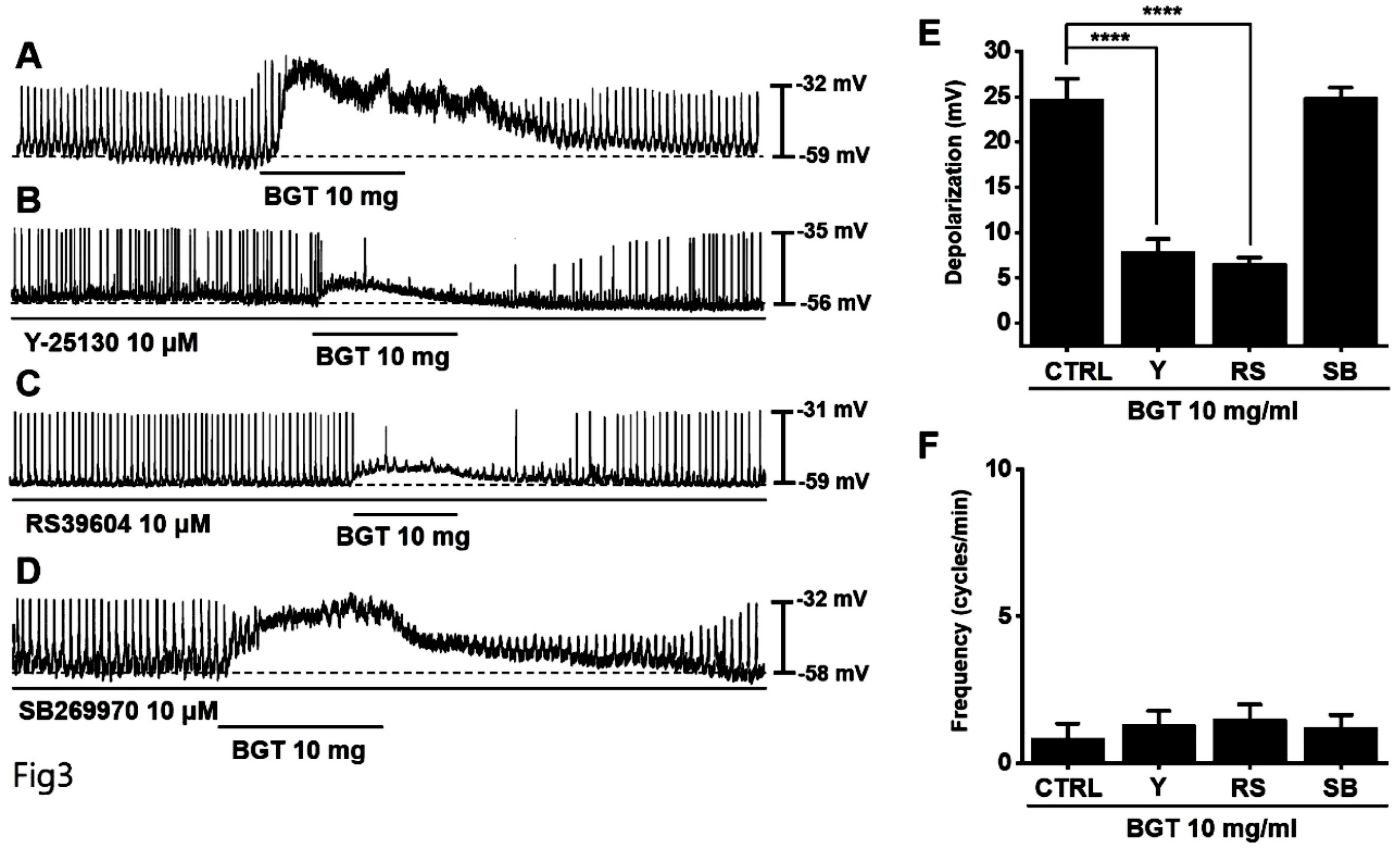
Effects of 5HTR antagonists on the ICC response to BGT. (A) 10 mg of BGT depolarized pacemaker potential and decreased frequency. (B) A 5HTR3 antagonist, Y25130, inhibited the ICC response to BGT. (C) RS39604, a 5HTR4 antagonist, inhibited the ICC reaction to BGT. (D) A 5HTR7 antagonist, SB269970, did not affect the ICC reaction to BGT. (E) Bar graph for depolarization change. (F) Bar graph for frequency change. Mean ± SEM. ****p < 0.0001. CTRL, control; Y, Y25130; RS, RS39604; SB, SB269970; BGT, *Bojunggunbi-tang*.

**Figure 4:**
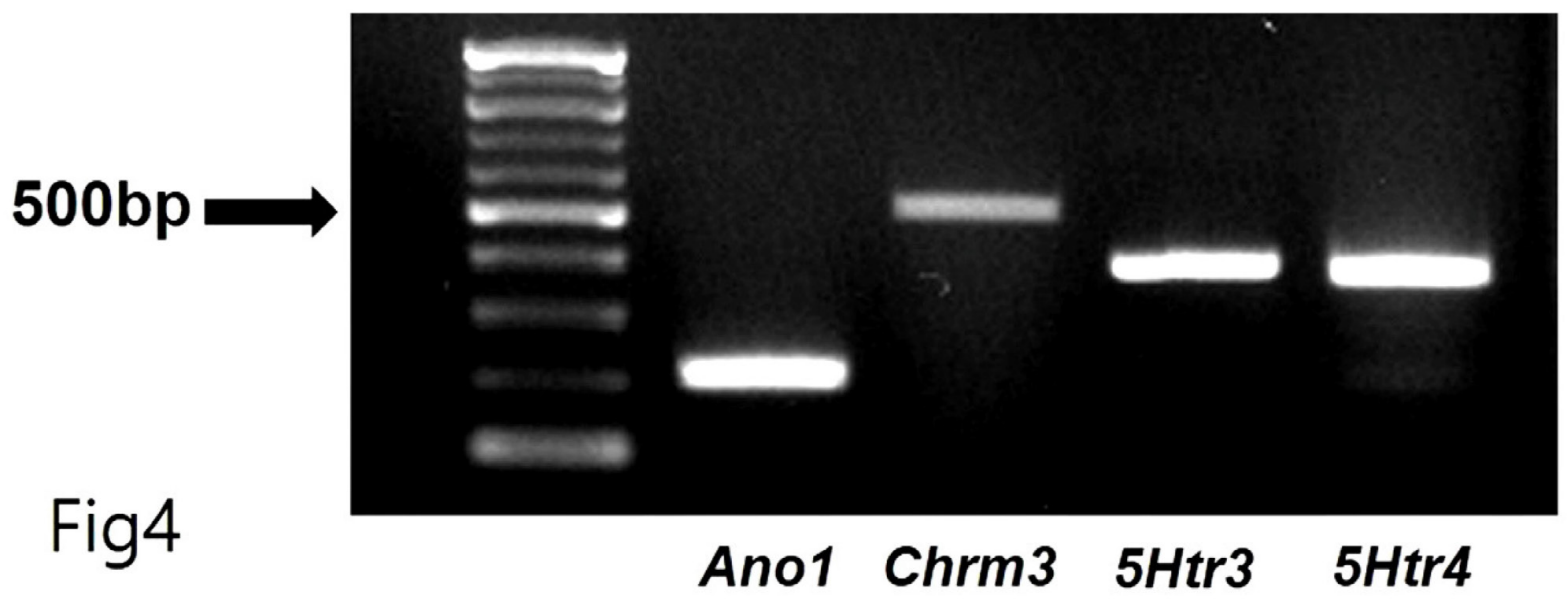
RT-PCR assay to find *Chrm3*, *5Htr3*, and *5Htr4* mRNA expressions in ICCs. RT-PCR detected the transcripts for *Ano1* (positive control, 213 base pairs), *Chrm3* (511 base pairs), *5Htr3* (399 base pairs), and *5Htr4* (359 base pairs) mRNA in ICCs.

Generally, 5-hydroxytryptamine receptors (5HTR) are involved in regulating GI motility 18,19, with ICCs expressing 5HTR3, 5HTR4, and 5HTR7 20,21. Therefore, we experimented with the respective antagonists. Pretreatment with Y-25130 and RS39604 (5HTR3 and 5HTR4 antagonists, respectively) inhibited the BGT-induced ICC response (Figs. 3A-C), whereas pretreatment with the 5HTR7 antagonist, SB269970, suppressed it (Fig. 3D). Additionally, Y-25130 pretreatment resulted in a depolarization of 7.9 ± 1.4 mV (*p* < 0.0001). In contrast, depolarization was 6.6 ± 0.7 mV (*p* < 0.0001) under RS39604 pretreatment and 24.9 ± 1.1 mV under SB269970 pretreatment (Fig. 3E). The frequency change was 1.3 ± 0.5 cycles/min, 1.5 ± 0.5 cycles/min, and 1.0 2 ± 0.4 cycles/min under Y-25130, RS39604, and SB269970 pretreatments, respectively (Fig. 3F). *Chrm3*, *5Htr3*, and *5Htr4* mRNA were detected in ICCs using RT-PCR (Fig. 4). These results indicated that BGT acted through CHRM3, 5HTR3, and 5HTR4.

### Involvement of Ca^2+^ and nonselective cation channels in ICC responses to BGT

**Figure 5:**
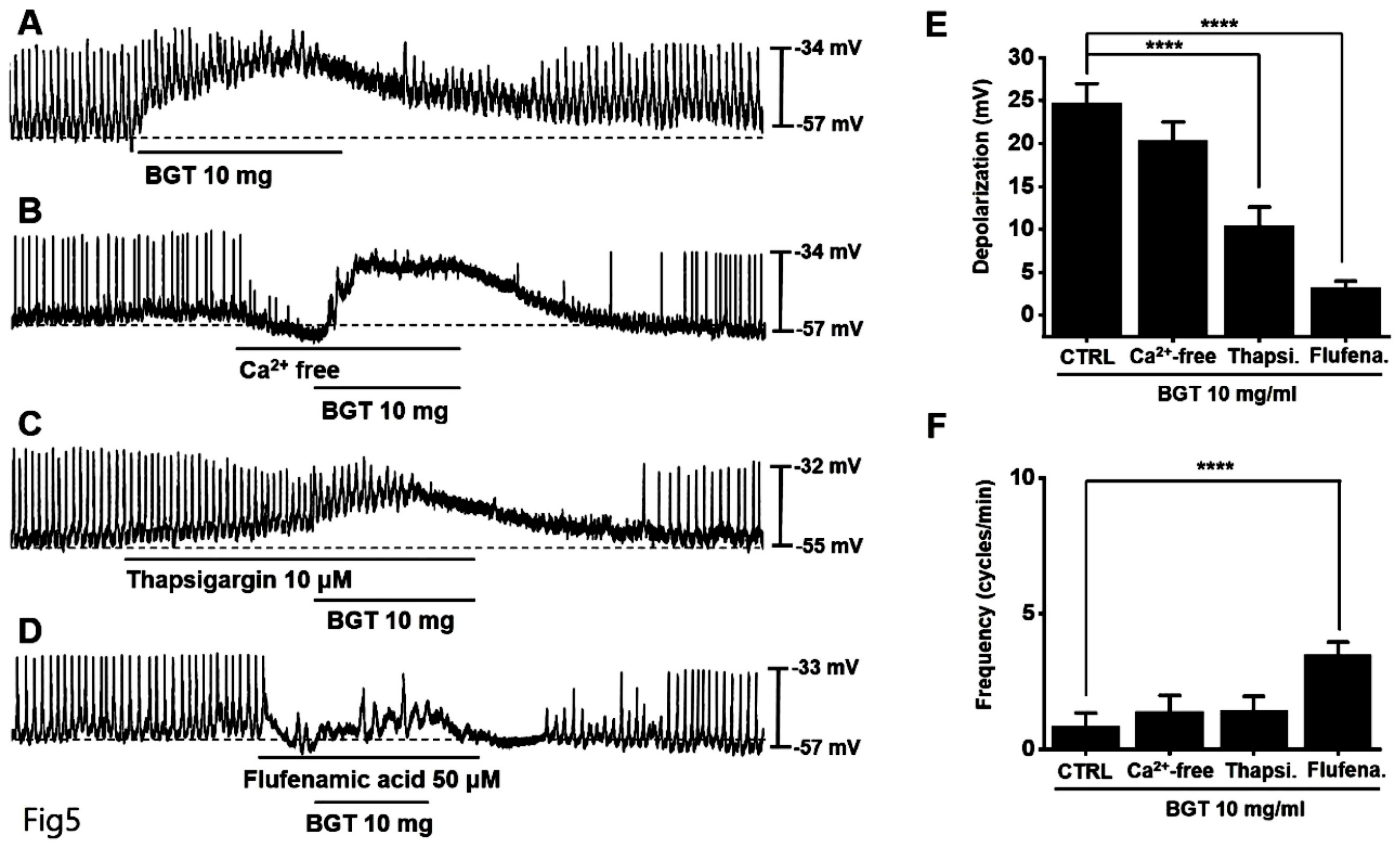
The involvement of Ca^2+^ and nonselective cation channels in the ICC response to BGT. (A) 10 mg of BGT depolarized pacemaker potential and decreased frequency. (B) Extracellular Ca^2+^ removal did not affect the ICC reaction to BGT. (C) Thapsigargin, an intracellular Ca^2+^ concentration regulator, inhibited the ICC reaction to BGT. (D) Flufenamic acid, a nonselective cation channel inhibitor, inhibited the ICC reaction to BGT. (E) Bar graph for depolarization change. (F) Bar graph for frequency change. Mean ± SEM. ****p < 0.0001. CTRL, control; Thapsi., Thapsigargin; Flufena., Flufenamic acid; BGT, *Bojunggunbi-tang*.

We removed extracellular Ca^2+^ to determine its role in the ICC response to BGT and observed that the removal did not affect the response (Fig. 5B). However, thapsigargin treatment decreased depolarization and frequency responses (Fig. 5C). Thapsigargin inhibits Ca^2+^ regulation in the sarcoplasmic reticulum, an intracellular Ca^2+^ regulatory organ.

Subsequently, we confirmed the relevance of nonselective cation channels in the cell membrane. We administered flufenamic acid, a nonselective cation channel inhibitor, and verified that it suppressed the BGT-induced response (Fig. 5D). Compared with the removal of extracellular Ca^2+^ (BGT-induced depolarization = 20.4 ± 2.2 mV), depolarization decreased to 10.4 ± 2.1 mV (*p* < 0.0001) under thapsigargin treatment and 3.2 ± 0.8 mV (*p* < 0.0001) under flufenamic acid treatment (Fig. 5E). Additionally, the BGT-induced frequency change was 1.4 ± 0.6 cycles/min upon extracellular Ca^2+^ removal. Under thapsigargin and flufenamic acid treatments, the frequency change was 1.4 ± 0.5 cycles/min and 3.5 ± 0.5 cycles/min (*p* < 0.0001), respectively (Fig. 5F). These results indicate that intracellular Ca^2+^ regulation and nonselective cation channels in the cell membrane are involved in the ICC response to BGT.

### Involvement of protein kinases in the ICC response to BGT

**Figure 6:**
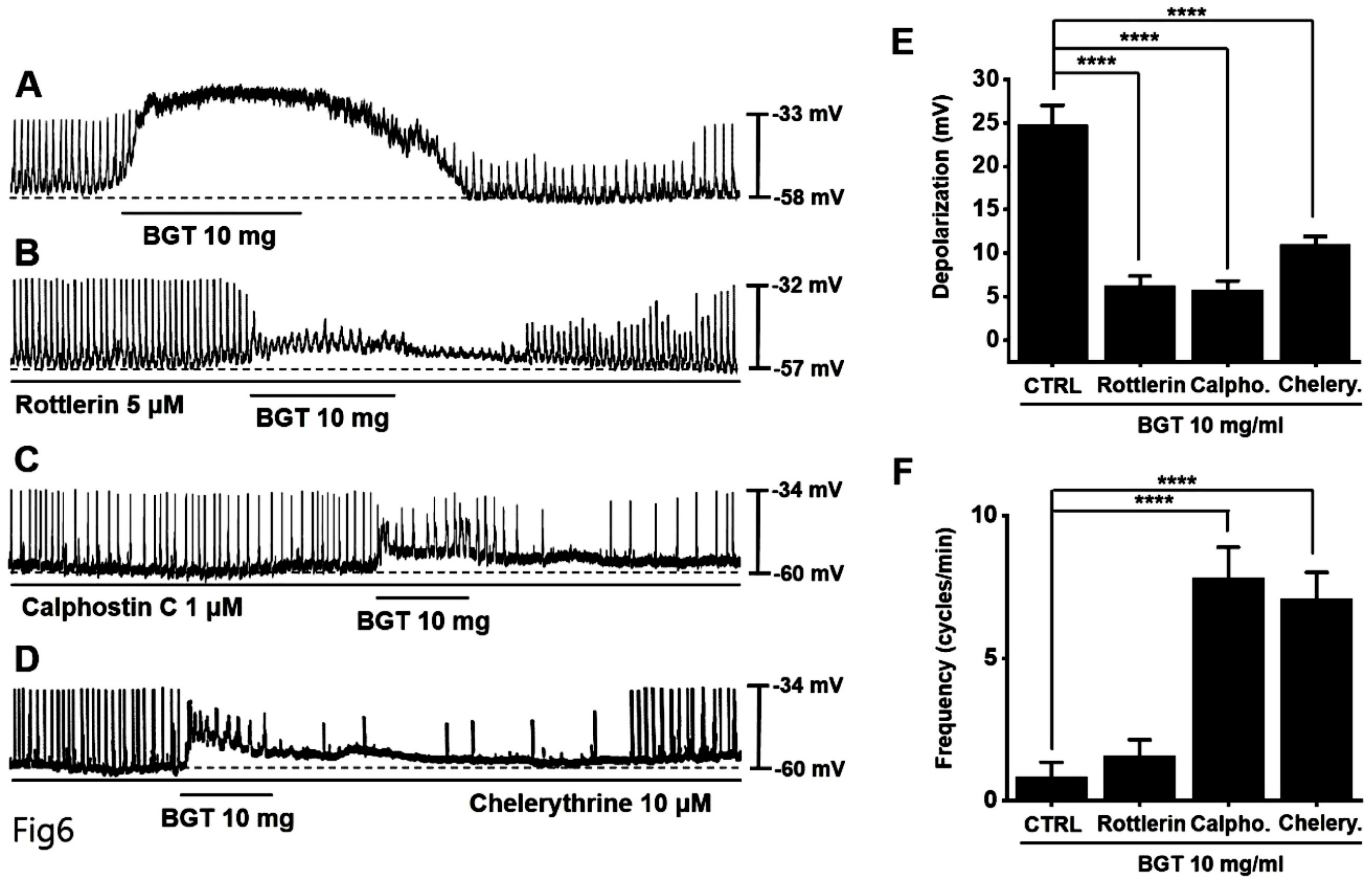
The involvement of PKC in the ICC response to BGT. (A) 10 mg of BGT depolarized pacemaker potential and decreased frequency. PKC inhibitors (B) Rottlerin, (C) Calpostin C, and (D) Cherrythrin inhibited the ICC response to BGT. (E) Bar graph for depolarization change. (F) Bar graph for frequency change. Mean ± SEM. ****p < 0.0001. CTRL, control; Calpho., Calphostin C; Chelery., Chelerythrine; BGT, *Bojunggunbi-tang*.

Next, we tested protein kinase C (PKC) and mitogen-activated protein kinase (MAPK) antagonists. Pretreatment with the PKC antagonists—rottlerin, calphostin C, and chelerythrine—inhibited the BGT-induced ICC response (Figs. 6A-D). Under rottlerin, calphostin C, and chelerythrine treatments, the depolarization values were 6.3 ± 1.1 mV (*p* < 0.0001), 5.8 ± 1.1 mV (*p* < 0.0001), and 10.9 ± 1.0 mV (*p* < 0.0001), respectively (Fig. 6E). Similarly, the frequency changes were 1.6 ± 0.6 cycles/min, 7.8 ± 1.1 cycles/min (*p* < 0.0001), and 7.1 ± 0.9 cycles/min (*p* < 0.0001) (Fig. 6F).

**Figure 7:**
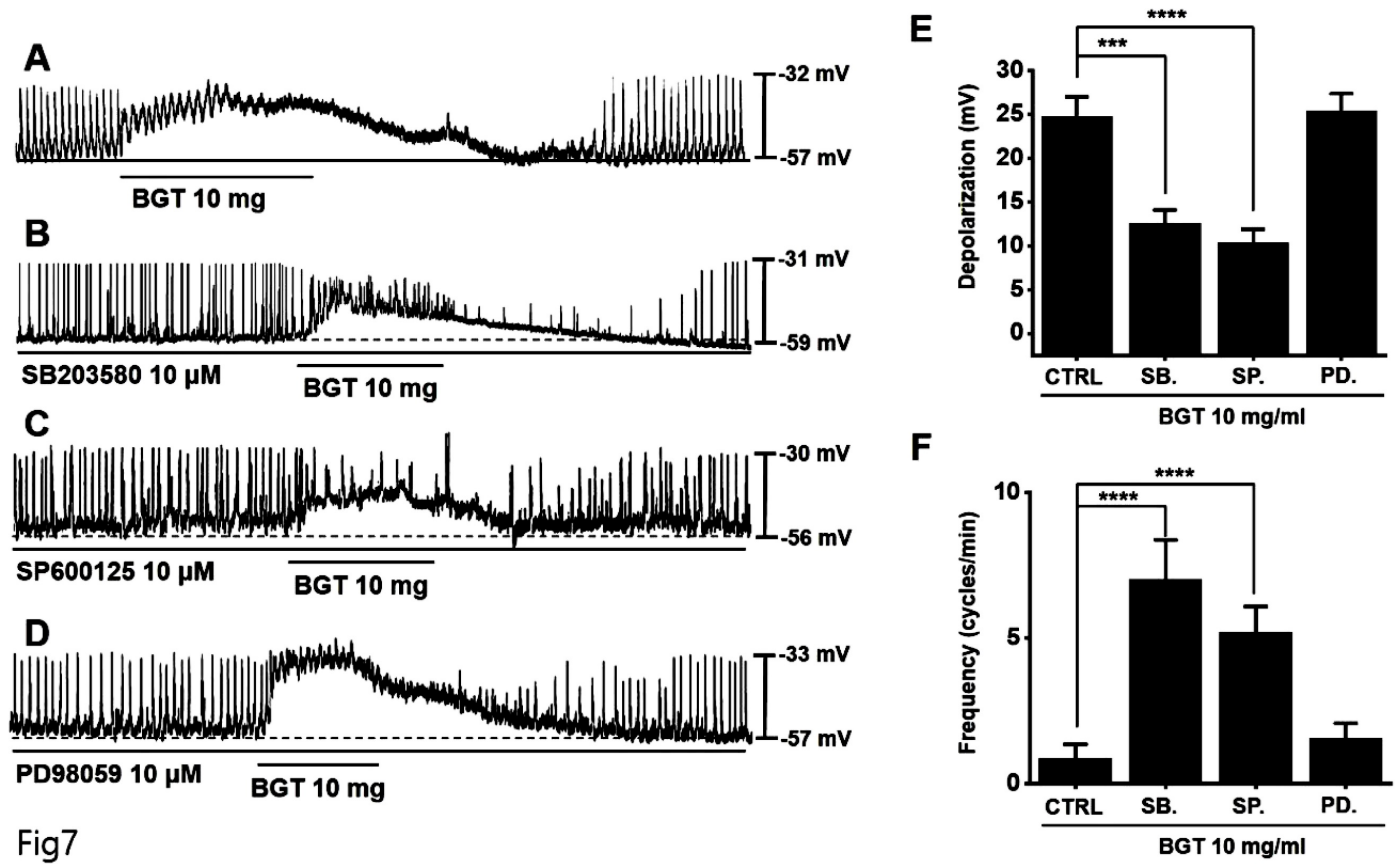
The involvement of MAPK in the ICC response to BGT. (A) 10 mg of BGT depolarized pacemaker potential and decreased frequency. MAPK inhibitors (B) SB203580 and (C) SP600125 inhibited the ICC response to BGT. (D) PD98059 did not affect the ICC reaction to BGT. (E) Bar graph for depolarization change. (F) Bar graph for frequency change. Mean ± SEM. ***p < 0.001. ****p < 0.0001. CTRL, control; SB., SB203580; SP., SP600125; PD., PD98059; BGT, *Bojunggunbi-tang*.

Next, pretreatment with the MAPK antagonists, SB203580 and SP600125, inhibited the ICC response to BGT, whereas pretreatment with the antagonist PD98059 did not have any effect (Fig. 7A-D). Under SB203580, SP600125, and PD98059, the depolarization values were 12.6 ± 1.5 mV (*p* < 0.001), 10.4 ± 1.5 mV (*p* < 0.0001), and 25.3 ± 2.1 mV, respectively (Fig. 7E). Furthermore, the frequency changes after treatment with the three MAPK antagonists were 7.0 ± 1.4 cycles/min (*p* < 0.0001), 5.2 ± 0.9 cycles/min (*p* < 0.0001), and 1.6 ± 0.5 cycles/min (Fig. 7F). These results indicate that the PKC and MAPK signaling pathways are involved in the ICC response to BGT.

### Involvement of guanylate cyclase and protein kinase G in the ICC response to BGT

**Figure 8:**
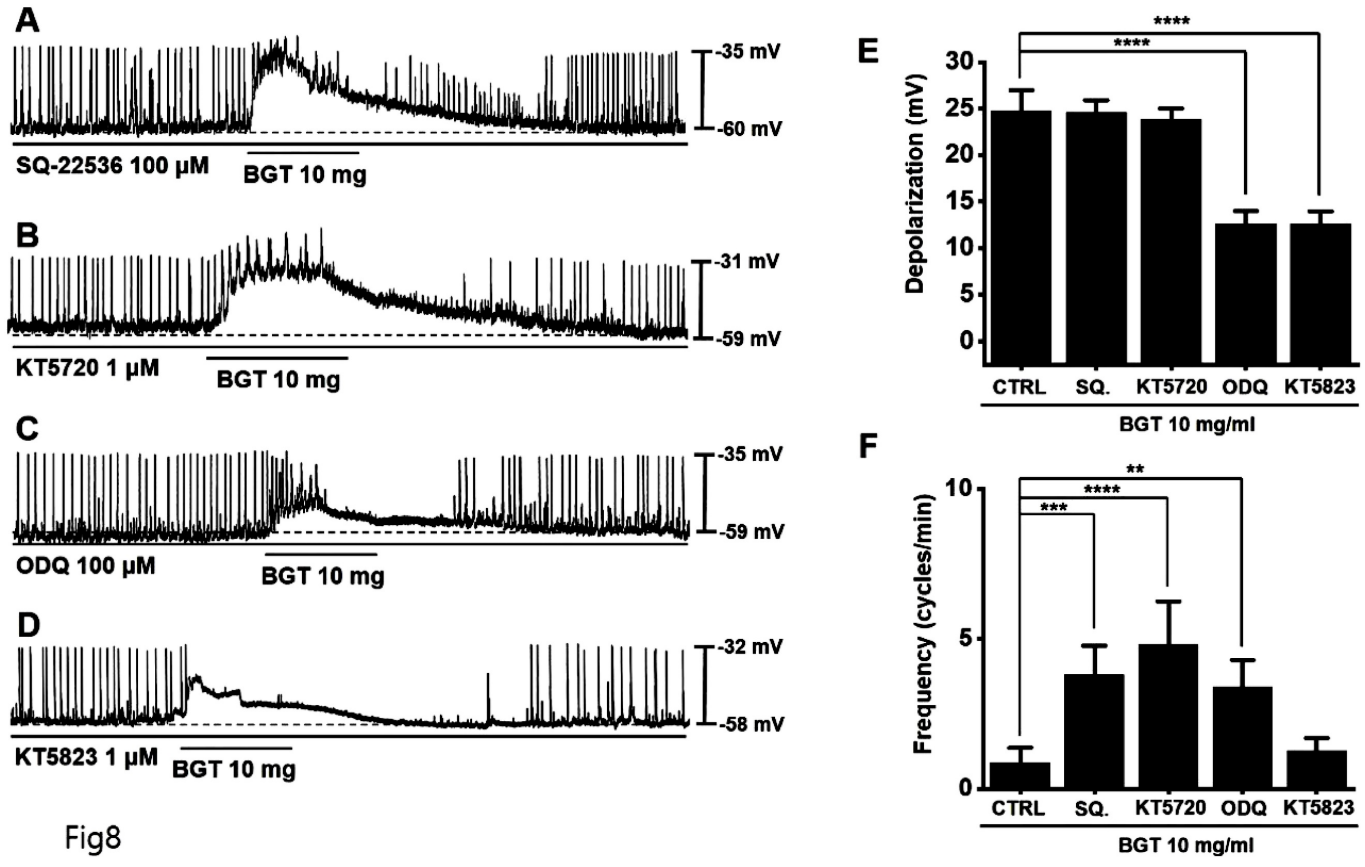
Involvement of adenylate cycle, guanylate cycle, PKA, and PKG in the ICC reaction to BGT. (A) SQ-22536, an adenylate cycle inhibitor, did not affect the ICC reaction to BGT. (B) KT5720, a PKA inhibitor, did not affect the ICC reaction to BGT. (C) ODQ, a guanylate cycle inhibitor, inhibited the ICC reaction to BGT. (D) KT5823, a PKG inhibitor, inhibited the ICC reaction to BGT. (E) Bar graph for depolarization change. (F) Bar graph for frequency change. Mean ± SEM. **p < 0.01. ***p < 0.001. ****p < 0.0001. CTRL, control; SQ., SQ22536; BGT, *Bojunggunbi-tang*.

We used the adenylate cyclase inhibitor SQ-22536 and the guanylate cyclase inhibitor ODQ to determine whether adenylate cyclase and guanylate cyclase are involved in BGT action. Pretreatment with SQ-22536 had no effect, although pretreatment with ODQ suppressed BGT action (Fig. 8A, C).

Additionally, we tested the protein kinase A (PKA) inhibitor, KT5720, and the protein kinase G (PKG) inhibitor, KT5823. Pretreatment with KT5720 had no effect, whereas pretreatment with KT5823 suppressed BGT action on ICCs (Figs. 8B, D). Under SQ-22536, KT5720, ODQ, and KT5823, the depolarization values were 24.6 ± 1.3 mV, 23.8 ± 1.2 mV, 12.6 ± 1.4 mV (*p* < 0.0001), and 12.5 ± 1.3 mV (*p* < 0.0001), respectively (Fig. 8E). Additionally, the frequency change was 3.8 ± 1.0 cycles/min (*p* < 0.001), 4.8 ± 1.5 cycles/min (*p* < 0.0001), 3.4 ± 0.9 cycles/min (*p* < 0.01), and 1.3 ± 0.4 cycles/min (Fig. 8F). Therefore, we determined that only the guanylate cyclase and PKG signaling pathways are involved in the BGT-induced response.

## Discussion

In traditional Korean medicine, BGT is widely prescribed for treating digestive diseases 11-13. Previous research has demonstrated that BGT has therapeutic efficacy against intestinal inflammation induced by dextran sulfate sodium and trinitrobenzene sulfonic acid 11,12. The medication can even protect the small intestine against wounds induced by nonsteroidal anti-inflammatory drugs 13. BGT has demonstrated effectiveness in treating abdominal pain, indigestion, and diarrhea 22. The prescription is a combination of “*Daehwajungeum*” and “*Sambaek-tang*,” two famous Korean medications for GI diseases, and is considered superior because it has no known side effects 22.

The GI tract comprises the mouth, pharynx, esophagus, stomach, small intestine, large intestine, and anus 23. It functions to digest food, absorb nutrients, and excrete waste 24. Well-coordinated GI motility is paramount for the successful physiological function of the GI tract, which involves regulating the speed of nutrient processing and absorption, as well as appetite and satiety control 25.

As pacemakers, ICCs are crucial for GI motility and are involved in neurotransmission 4-8. Recent breakthroughs have been made in understanding the ICC activation mechanism, including the diversification of research methods 1. Future studies are expected to focus on the relationship between ICCs and GI-motility diseases, particularly the development of techniques or drugs that can control ICC function internally and externally.

Here, we confirmed that BGT depolarized and reduced the pacemaker-potential frequency in ICCs (Fig. 1). We also determined that BGT acted through CHRM3, 5HTR3, and 5HTR4 (Figs. 2-4). Furthermore, extracellular Ca^2+^ was uninvolved in BGT efficacy, whereas intracellular Ca^2+^ and nonselective cation channels in the cell membrane were involved (Fig. 5). Antagonists were used to successfully identify the signaling pathways involved in the BGT response. Specifically, the PKC, MAPK, guanylate cycle, and PKG signaling pathways were all involved in the BGT response (Figs. 6-8). Therefore, we can confirm that BGT regulates ICCs and GI motility through these pathways. In the future, we recommend *in vivo* experiments to explore the possibility of controlling GI motility through these pathways. Previous studies have attributed BT efficacy against colitis to the effects of a combination of well-known herbs, including *Lonicera japonica*, *Crataegus pinnatifida*, and *Glycyrrhiza uralensis* 26-28. Thus, in a future study, we plan to determine which of the 16 herbs in BGT exerts the strongest influence on ICCs and GI motility control and eventually identify the active ingredients.

CHRM3 is present throughout the body, including in smooth muscles, endocrine glands, exocrine glands, lungs, intestines, and the brain 29. This receptor causes smooth muscle contraction and gland secretion 29 and is paired with Gq proteins, which activate phospholipase C to alter IP_3_ and intracellular calcium concentrations 29. Furthermore, CHRM3 is associated with the PKC and MAPK pathways 30. CHRM3 regulates the cardiovascular system in rats by controlling the guanylate cycle through nitric oxide 31.

5HTR is a group of G protein-binding receptors and ligand-opening ion channels in the central and peripheral nervous systems 32 that mediate excitatory and inhibitory neurotransmission 33. Activated by the neurotransmitter serotonin, serotonin receptors control the release of other neurotransmitters—including glutamate, GABA, dopamine, epinephrine, norepinephrine, and acetylcholine—and hormones, such as oxytocin, prolactin, vasopressin, and corticotropin 34. Hence, serotonin receptors affect many biological and neurological processes, including aggression, anxiety, appetite, cognition, learning, memory, mood, nausea, sleep, and body temperature 34. They are the target of many pharmaceutical and mood-altering drugs, including antidepressants, antipsychotics, anorexics, and hallucinogens. 5HTR3 is the only serotonin receptor known to regulate the ionic channel, which, in turn, controls the ligand-gated Na^+^ and K^+^ cation channels. Another important serotonin receptor class is 5HTR4, which increases cAMP concentration by binding to G-proteins 35. However, some reports have suggested that 5HTR3 may also increase cGMP 36.

**Figure 9:**
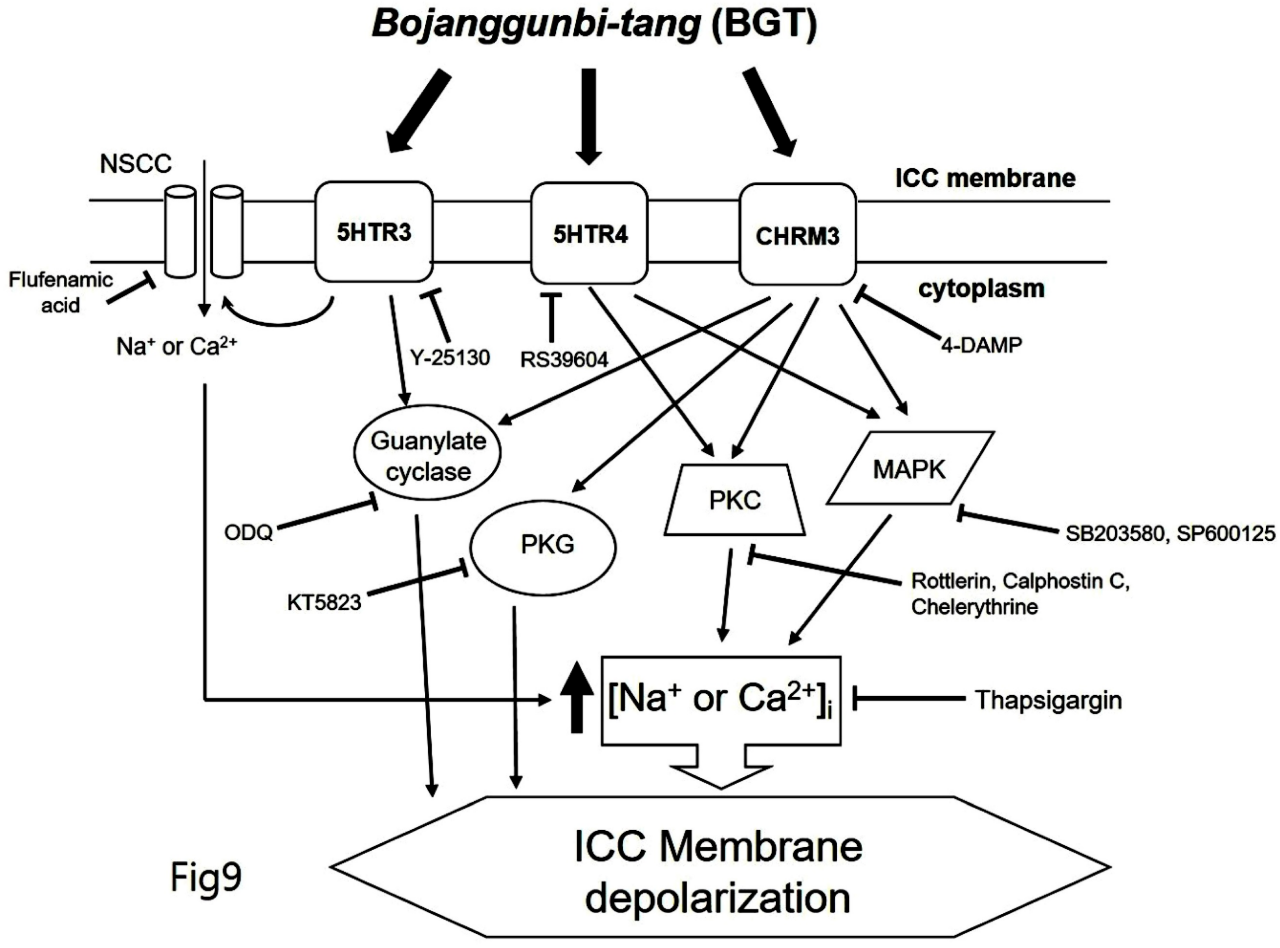
Hypothetical schematic signaling pathway of BGT-induced ICC depolarization. CHRM3, 5HTR3, and 5HTR4 seemingly mediate BGT-induced ICC depolarization to regulate the intracellular calcium, PKC, MAPK, guanylate cycle, and PKG mechanisms to exhibit a response.

We demonstrated that BGT acts on CHRM3, 5HTR3, and 5HTR4 to regulate intracellular calcium through the PKC, MAPK, guanylate cycle, and PKG mechanisms. Based on this study's findings, we propose the following model of BGT effects on ICCs (Fig. 9). In particular, CHRM3 is believed to be involved in the intracellular calcium, PKC, MAPK, guanylate, and PKG pathways, while 5HTR is involved in relatively few pathways (intracellular calcium, PKC, and MAPK). A difficult aspect of traditional medicine research is that the actual mechanisms of a given effect often differ from well-known explanations. However, identifying a novel mechanism provides an important advantage in uncovering new treatments from old prescriptions. Traditional medicine research remains an unexplored field with great potential for unexpected therapeutic discoveries.

Traditional medicine is especially useful for functional diseases and those who experience adverse side effects from Western treatments or find them ineffective due to constitutional problems. In the early stages of organic disease, traditional medication may be more effective than Western treatment while also being effective against chronic and consumable diseases. Most GI-motility disorders are functional diseases and thus potentially amenable to traditional medicine, particularly when considering the evidence of therapeutic efficacy without side effects. In conclusion, we believe that BGT is a safe and effective treatment for controlling GI motility.
